# Data supporting the design and evaluation of a universal primer pair for pseudogene-free amplification of HPRT1 in real-time PCR

**DOI:** 10.1016/j.dib.2015.06.009

**Published:** 2015-07-06

**Authors:** Reza Valadan, Akbar Hedayatizadeh-Omran, Mahdyieh Naghavi Alhosseini-Abyazani, Omolbanin Amjadi, Alireza Rafiei, Mohsen Tehrani, Reza Alizadeh-Navaei

**Affiliations:** a Molecular and Cell Biology Research Center (MCBRC), Department of Immunology, Faculty of Medicine, Mazandaran University of Medical Sciences, Sari, Mazandaran, Iran; b Department of Immunology, Faculty of Medicine, Mazandaran University of Medical Sciences, Sari, Mazandaran, Iran

## Abstract

Hypoxanthine-guanine phosphoribosyltransferase 1 (*HPRT1*) is a common housekeeping gene for sample normalization in the quantitative reverse transcriptase polymerase chain (qRT-PCR). However, co-amplification of *HPRT1* pseudogenes may affect accurate results obtained in qRT-PCR. We designed a primer pair (HPSF) for pseudogene-free amplification of *HPRT1* in qRT-PCR [1]. We showed specific amplification of HPRT1 mRNA in some common laboratory cell lines, including HeLa, NIH/3T3, CHO, BHK, COS-7 and VERO. This article provides data supporting the presence and location of *HPRT1* pseudogenes within human and mouse genome, and the strategies used for designing primers that avoid the co-amplification of contaminating pseudogenes in qRT-PCR. *In silico* analysis of human genome showed three homologous sequences for *HPRT1* on chromosomes 4, 5 and 11. The mRNA sequence of HPRT1 was aligned with the pseudogenes, and the primers were designed toward 5′ end of HPRT1 mRNA that was only specific to HPRT1 mRNA not to the pseudogenes. The standard curve plot generated by HPSF primers showed the correlation coefficient of 0.999 and the reaction efficiency of 99.5%. Our findings suggest that HPSF primers can be recommended as a candidate primer pair for accurate and reproducible qRT-PCR assays.

**Table:** 

**Subject area**	Biology
**More specific subject area**	Molecular biology, Quantitative real time PCR
**Type of data**	Table, figure
**How data was acquired**	*In silico* analysis of *HPRT1* pseudogenes using online bioinformatics tools and CLC Main Workbench software (Qiagen, USA) and Allele ID primer design software version 7.5 (Premier Biosoft, USA).Primer efficiency data acquired by analysis of amplification curve using quantitative real time PCR (iQ5, Biorad,USA)
**Data format**	Raw, analyzed
**Experimental factors**	*HPRT1* pseudogenes, *HPRT1* expression, standard curve, *HPRT1* expression in HeLa, NIH/3T3, CHO, BHK, VERO and COS-7
**Experimental features**	Bioinformatics analysis of human and mouse genome were performed to find *HPRT1* pseudogenes. HPRT1-specific primers were designed and tested for pseudogene-free amplification of *HPRT1* in different cell lines. The primers specificity and efficiency were also determined in qRT-PCR.
**Data source location**	Sari, Iran
**Data accessibility**	Data is provided with this and the main article [1].

**Value of the data**

• *HPRT1* is a sensitive internal control for normalization of gene expression in qRT-PCR.
• HPSF primer pair allows pseudogene-free amplification of HPRT1 mRNA in qRT-PCR.
• HPSF primer pair provides specific and high efficient amplification of *HPRT1* in qRT-PCR.
• HPSF primer pair amplifies HPRT1 mRNA across a wide range of mammalian species.

## 1 Data, experimental design, materials and methods

### 1.1 Primer design

**Fig. 1:**
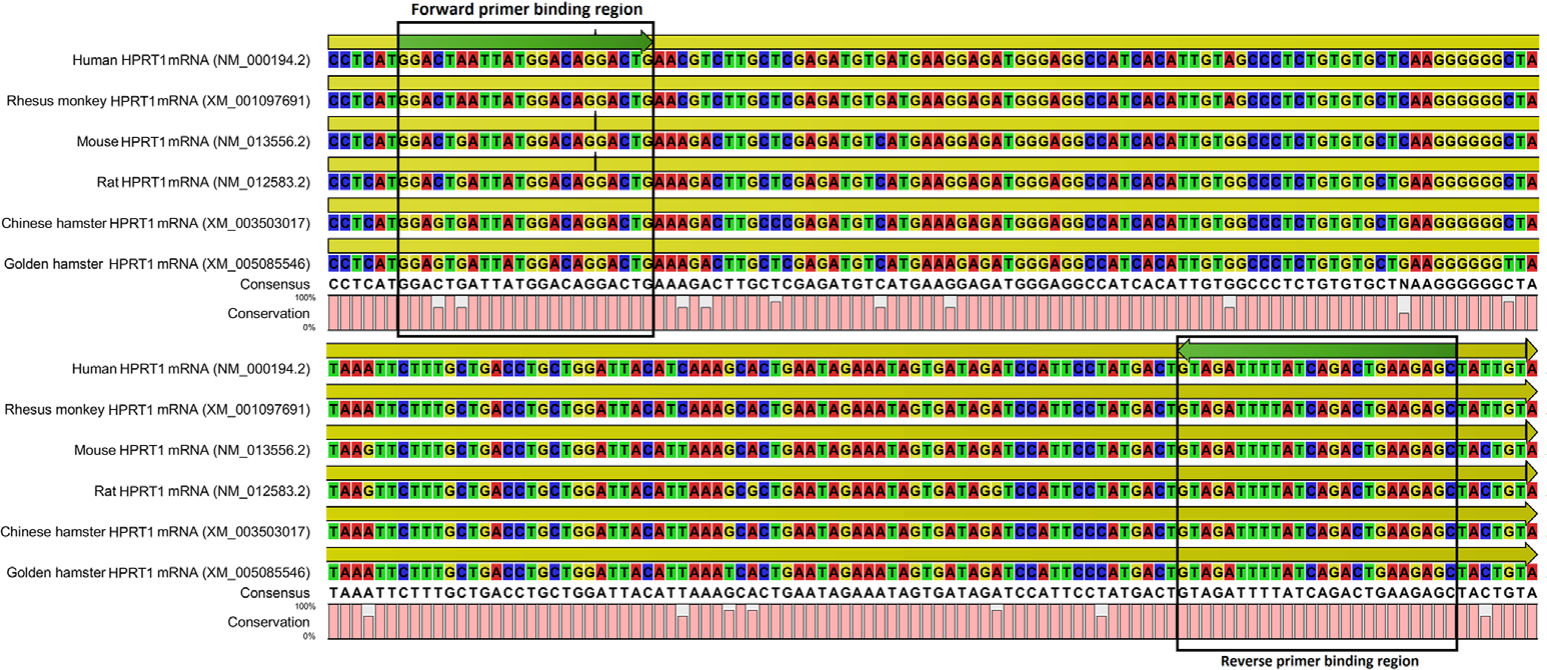
Alignment of HPRT1 mRNAs from different species to identify appropriate regions for primer design. High level of sequence similarity between HPRT1 mRNA sequences in different species makes it possible to design a universal cross-species primer. Black boxes show forward and reverse primer binding regions.

Prior to the identification of human genome sequences, it was shown that *HPRT1* contained four pseudogenes on chromosome 3, 5, and 11 [2,3]. Here to identify putative pseudogenes of *HPRT1*, human and mouse HPRT1 mRNA sequences were used as baits in the Blat and Blast online tools at UCSC Genome Bioinformatics (https://genome.ucsc.edu/) and National Centre for Biotechnology Information (http://www.ncbi.nlm.nih.gov/) respectively. Sequences scored more than 400 and 283 for human and mouse HPRT1 mRNA were selected for further analysis. In consistent with a previous study [4], we also identified three pseudogenes for human *HPRT1* on chromosomes 4, 5, and 11 and one for mouse *HPRT1* on chromosome 17 (Table 1). To design pseudogene-free primers, HPRT1 mRNA and pseudogene sequences were aligned using CLC Main Workbench software version 5.5 (Qiagen, USA) (see Fig. 1 in Ref. [1]). In order to avoid co-amplification of genomic DAN (gDNA) contamination, unique regions of HPRT1 mRNA that corresponded to exon−exon junctions were selected. In addition, HPRT1 mRNA sequences of human, mouse, rat, Chinese hamster, golden hamster, and rhesus monkey were aligned to design a universal primer pair capable of amplifying HPRT1 mRNA in these species (Fig. 1). Primers were designed using Allele ID primer design software version 7.5 (Premier Biosoft, USA) (Table 2). *In silico* analysis also demonstrated that HPSF primer pair potentially covers a wide taxonomic range in mammals spanning from whale to human (Table 3).

### 1.2 Cell lines and cell culture

HeLa, NIH/3T3, CHO, BHK, VERO and COS-7 cell lines were obtained from Pasteur Institute of Iran (Tehran, Iran). The cells were cultured in RPMI 1640 (PAA, Pasching/Austria) containing inactivated fetal bovine serum (PAA, Pasching/ Austria), l-glutamine (300 mg/l), penicillin (100 U/ml) and streptomycin (100 μg/ml). The cell lines were maintained in 5% CO_2_ at 37 °C in an incubator (Memmert, Germany) with 95% humidity.

### 1.3 Nucleic acid extraction

The cell lines were cultured to Subconfluent stage in 25 cm^2^ culture flasks (SPL, South Korea) and then genomic DNA and total RNA was extracted using AccuPrep^®^Genomic DNA Extraction Kit (Bioneer, Korea) and Qiagen RNeasy Mini Kit (Qiagen, Germany), respectively. The quantity and integrity of the isolated nucleic acids were verified by Nano-spectrophotometer (WPA, UK) and electrophoresis in a 2% agarose gel, respectively.

### 1.4 First strand cDNA synthesis and polymerase chain reaction

Complementary DNA (cDNA) was synthesized from 1 microgram of the total RNA in a 20 µl reaction using the Omniscript-RT-Kit (Qiagen, Germany) according to manufacturer’s instruction. PCR reaction was carried out in a total volume of 20 µl containing of 10 mM Tris HCl pH 8.4, 50 mM KCl, 200 nmol each forward and reverse primers, 1.5 mM MgCl_2_, 250 µM dNTP, 1 U of Taq DNA polymerase (Thermo Scientific, Germeny), 2 µl cDNA and 100 ng DNA as templates. The thermal profile included an initial denaturation at 94 °C for 2 min, followed by 40 cycles of amplification at 94 °C for 30 s, 60 °C for 30 s and 72 °C for 30 s and a final extension step at 72 °C for 5 min. The reactions were cycled in an Eppendorf MasterCycler gradient thermal cycler (Germany). The PCR products were visualized after separation on a 2% agarose gel and staining with ethidium bromide. The results of PCR in the all tested cell lines showed that HPSF primers amplified the expected 195 bp products only when cDNA used as templates (see Fig. 1 in Ref. [1]).

### 1.5 DNA sequencing

The accuracy of the amplification reaction was validated by DNA sequencing of the PCR products. The human and mouse amplicons were excised from a 2% agarose gel, and purified using the QIAquick Gel Extraction Kit (Qiagen, Germany) according to the manufacturer’s instructions. The quantity of the purified plasmid was then measured by spectrophotometer (Biochrom WPA, UK). About 10 ng of each amplicon was ligated into pTG19-T cloning vector (Vivantis, Malaysia) and transformed into chemically competent bacterial cells. Sequencing of the inserts was performed using capillary DNA analyzer (ABI 3730, Applied Biosystems, USA) after sequencing reactions with a Big Dye Terminator V3.1 Cycle Sequencing Kit (Applied Biosystems). The nucleotide sequences of human and mouse HPRT1 mRNA were submitted to the GenBank database under the accession numbers KR817914 and KR817915, respectively.

### 1.6 Real-time PCR

**Fig. 2:**
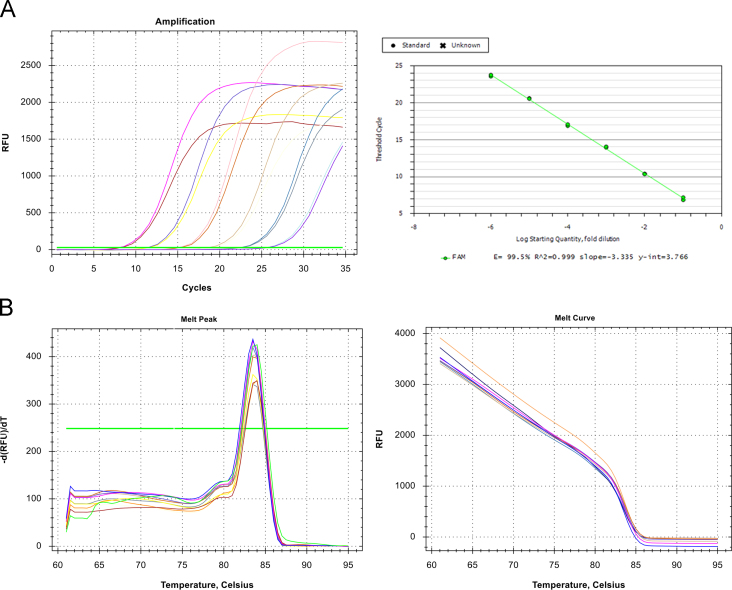
Representative amplification plots obtained from a 10-fold serial dilution. The corresponding standard curve showed a slope of line −3.335, efficiency 99.5% and R2 0.999 (A). Melting curves and melting peaks of the serial dilution confirmed a specific amplification (B).

Real-time PCR reactions were performed using 2X Thermo Scientific Maxima SYBR Green/ROX qPCR Master Mix (Fisher Scientific, Germany) and run in an iCycler iQ5 (Bio RAD, USA) instrument. The reaction was carried out in a total volume of 20 µl containing 10 µl of 2X master mix, 200 nmol of each forward and reverse primer, and 2 µl of 10-fold serially diluted of HeLa cDNA or pTG19/*HPRT1* plasmid as templates. Cycling condition involved an enzyme activation step at 95 °C for 10 min followed by 40 cycles of 95 °C for 10 s, 60 °C for 15 s, and 72 °C for 15 s. In each cycle, an extra step of 72 °C for 10 s was included to collect fluorescence. At the end of PCR, to evaluate specific amplification of the target genes, melting curves ranging from 60 to 95 °C were also included in each run. The slope, intercept, amplification efficiency, and correlation coefficient (*R*^2^) of the primer pair were calculated as described previously [5]. The regression line had the correlation coefficient (*R*^2^ value) of 0.999 and the reaction efficiency was calculated 99.5% that confirmed accurate real-time results (Fig. 2A). The melt curve analysis also confirmed the specific amplification of HPRT1 mRNA with no evidence for primer dimer or nonspecific products (Fig. 2B). The importance of pseudogene-free amplification of the housekeeping gene in qRT-PCR has been emphasized for glyceraldehyde-3-phosphate dehydrogenase [6], and the data provided here also indicate that primers should be carefully selected for *HPRT1* to ensure accurate transcripts quantification in qRT-PCR.

## Figures and Tables

**Table 1:** Detail information of HPSF primers.

**Primer name**	**Primer sequences (5′−3′)**	**Length (bp)**	***T***_***m***_**(**°**C)**	**GC content (%)**	**Nucleotide position (NM_000194.2)**
**HPSF -F**	GGACTAATTATGGACAGGACTG	22	61.8	45.5	285–306
**HPSF -R**	GCTCTTCAGTCTGATAAAATCTAC	24	61	37.50	456–479

**Table 2:** *HPRT1* pseudogenes in human and mouse.

***HPRT1*****pseudogenes (NCBI gene ID)**	**Chromosomal location**	**Identity to HPRT1 mRNA (% and positions)**
***HPRT1P1*****(ID: 100130067)**	Chromosome 4, NC_000004.12 (15864937. 15865569 complement)	75%
		(237–559) (549–1352)
***HPRT1P2*****(ID: 3254)**	Chromosome 5, NC_000005.10 (30248374. 30249481)	77%
		(55–669) (836–1413)
***HPRT1P3*****(ID: 3255)**	Chromosome 11, NC_000011.10 (93990676. 93991522, complement); Chromosome 11, NC_000011.10 (93998649. 93999220, complement)	83%
		(162–725) (748–1377)
***Hprt-ps1*****(ID: 111269)**	Mouse Chromosome 17, NC_000083.6 (65396008. 65395223 complement)	75%
		(526–1272)

**Table 3:** HPRT1 mRNA sequences can be amplified by HPSF primer in various species.

***Species***^a^	**Accession number**	***Species***^b^	**Accession number**	***Species***^c^	**Accession number**
***Homo sapiens***	NM_000194.2	*Neovison vison*	JN587807.1	*Microtus rossiaemeridionalis*	GU645978.1
***Tupaia chinensis***	XM_006171444.1	*Mirounga angustirostris*	JN820130.1	*Microtus arvalis*	GU645977.1
***Spermophilus tridecemlineatus***	XM_005337680.1	*Loxodonta Africana*	XM_003420864.1	*Myodes glareolus*	JN701897.1
***Echinops telfairi***	XM_004715363.1	*Bos Taurus*	NM_001034035.2	*Microtus oeconomus*	JN701898.1
***Jaculus jaculus***	XM_004653986.1	*Mustela putoriusfuro*	XM_004815015.1 XM_004778420.1	*Cricetulus longicaudatus*	X59652.1
					X17656.1
***Tursiops truncatus***	XM_004323200.1	*Bos mutus*	XM_005911180.1	*Ochotona princeps*	XM_004582998.1
***Orcinus orca***	XM_004285480.1	*Leptonychotes weddellii*	XM_006744882.1	*Microtus ochrogaster*	XM_005358462.1
			XM_006730753.1		
***Nomascus leucogenys***	XM_003272534.2 XM_003272533.2	*Bubalus bubalis*	XM_006074802.1	*Cricetulus griseus*	XM_003503017.1
***Gorilla gorilla gorilla***	XM_004064891.1	*Panthera tigrisaltaica*	XM_007093584.1	*Mus spretus*	M20011.1
***Saimiri boliviensisboliviensis***	XM_003931150.1	*Felis catus*	XM_006944016.1	*Mesocricetu sauratus*	XM_005085546.1
***Papio Anubis***	XM_003918303.1	*Bubalus bubalis*	XM_006074802.1	*Mus musculus*	NM_013556.2
***Pan paniscus***	XM_003814508.1	*Panthera tigrisaltaica*	XM_007093584.1	*Rattus norvegicus*	NM_012583.2
***Otolemur garnettii***	XM_003796967.1	*Trichechus manatus latirostris*	XM_004379641.1	–	–
***Pongo abelii***	XM_002832128.2	*Ovis aries*	XM_004022693.1	–	–
***Callithrix jacchus***	XM_002763292.2	*Myotis davidii*	XM_006759719.1	–	–
***Macaca mulatta***	XM_001097691.2	*Vicugna pacos*	XM_006215984.1 XM_006201457.1	–	–
***Elephantulus edwardii***	XM_006881880.1	*Camelus ferus*	XM_006194437.1	–	–
***Sus scrofa***	NM_001032376.2	*Myotis lucifugus*	XM_006082071.1	–	–
***Macaca fascicularis***	NM_001283594.1	*Pantholops hodgsonii*	XM_005982399.1 XM_005956708.1	–	–
***Pan troglodytes***	NM_001110817.1	*Myotis brandtii*	XM_005872845.1	–	–
***Tursiops truncatus***	DQ404543.1	*Capra hircus*	XM_005700316.1 XM_005698763.1	–	–
***Stenella coeruleoalba***	DQ533610.1	Lipotes vexillifer	XM_007462883.1	–	–
***Akodon cursor***	AF254384.1	*Balaenoptera acutorostrata scammoni*	XM_007174545.1	–	–
–	–	*Physeter catodon*	XM_007117444.1 XM_007101314.1	–	–

a The HPSF primer set is fully matched with these species.

b HPSF forward primer contains a mismatch at position of 21 for these species.

c HPSF forward primer contains a mismatch at position of 6 for these species.
